# Laser-Induced Graphene: En Route to Smart Sensing

**DOI:** 10.1007/s40820-020-00496-0

**Published:** 2020-08-03

**Authors:** Libei Huang, Jianjun Su, Yun Song, Ruquan Ye

**Affiliations:** 1 Department of Chemistry, City University of Hong Kong, Kowloon, Hong Kong People’s Republic of China; 2 State Key Lab of Marine Pollution, City University of Hong Kong, Kowloon, Hong Kong People’s Republic of China

**Keywords:** Laser-induced graphene, Smart sensor, Printable electronics, Design principle

## Abstract

The discovery of laser-induced graphene (LIG) from polymers in 2014 has aroused much attention in recent years. A broad range of applications, including batteries, catalysis, sterilization, and separation, have been explored. The advantages of LIG technology over conventional graphene synthesis methods are conspicuous, which include designable patterning, environmental friendliness, tunable compositions, and controllable morphologies. In addition, LIG possesses high porosity, great flexibility, and mechanical robustness, and excellent electric and thermal conductivity. The patternable and printable manufacturing process and the advantageous properties of LIG illuminate a new pathway for developing miniaturized graphene devices. Its use in sensing applications has grown swiftly from a single detection component to an integrated smart detection system. In this minireview, we start with the introduction of synthetic efforts related to the fabrication of LIG sensors. Then, we highlight the achievement of LIG sensors for the detection of a diversity of stimuli with a focus on the design principle and working mechanism. Future development of the techniques toward in situ and smart detection of multiple stimuli in widespread applications will be discussed.

## Highlights

- Summarizing the strategies for the synthesis and engineering of laser-induced graphene, which is essential for the design of high-performance sensors.
- Introducing LIG sensors for the detection of various stimuli with a focus on the design principle and working mechanism.
- Discussing the integration of LIG sensors with signal transducers and conveying the prospects of smarting sensing systems to come.

## Graphical abstract

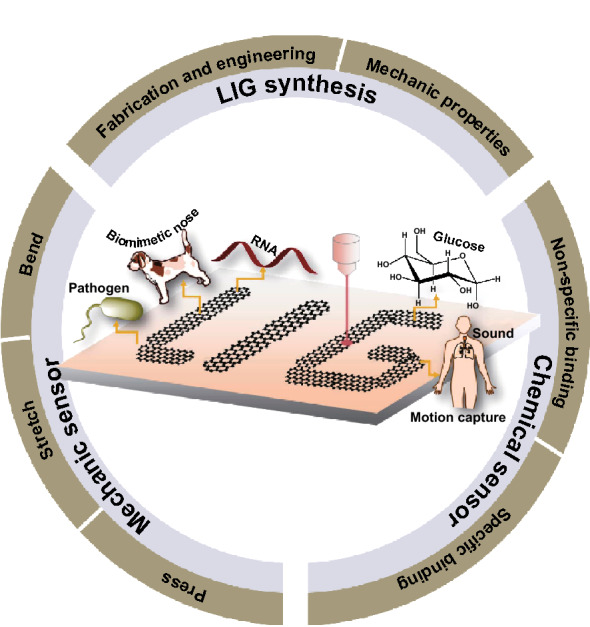

## Introduction

The report of high electron mobility and stability of high-quality few-layer graphene exfoliated by the “Scotch Tape” in 2004 was reputed a groundbreaking experiment in materials science [1]. Since then, many researchers have been devoted to exploring its fundamental properties and developing applications in broad fields. To commercialize graphene, various synthesis protocols have been developed, such as mechanical exfoliation, chemical vapor deposition, and chemical reduction of graphene oxide [2]. These methods have the advantage in manufacturing graphene of different grades, yet their scale-up productions could be hampered by the weakness such as low productivity, high energy consumption, and massive wastes generation. In 2014, it was found that polymers such as polyimide (PI) could be directly converted to porous graphene using an infrared CO_2_ laser, a machine that is commonly found in industry [3]. Besides infrared CO_2_ (10.6 μm) laser, visible laser [4–9] and ultraviolet laser [10] have also been successfully used to synthesize LIG. For infrared laser, the photothermal effect was suggested to account for the transition. Under instantaneous pyrolysis, the chemical bonds in the precursor would be broken and recombined with the release of gas [3]. For ultraviolet laser, a photochemical process was more likely to happen. Since the photo energy of ultraviolet laser is close to that of chemical bonds, it could directly break the chemical bonds in precursor and generate LIG [10]. For visible laser, both photothermal effect and photochemical effect contribute to the LIG formation [4, 9]. The laser irradiation process was performed in ambient conditions with miniscule wastes generated. In addition, the shape of LIG could be easily controlled by the computer design, which holds a great promise toward the development of printable electronics. The LIG has a surface area of 428 m^2^ g^−1^ and resistance of ≤ 10 Ω/□ [11, 12], which are comparable to the graphene synthesized by the conventional methods. Prior to LIG technology, many manufacturing methods that are also patternable have been developed to fabricate graphene, such as screen printing [13–15], 3D printing [16–21], and photolithography [22–24]. Table 1 analyzes these technologies’ merits and flaws. The unique advantages of LIG have made it a popular graphene synthesis method nowadays.

**Table 1:** Comparison of screen printing, 3D printing, photolithography, and LIG

	Screen printing	3D printing	Photolithography	LIG
Patternable	✓	✓	✓	✓
Mask/mold-free	×	×	×	✓
High resolution	✓ (40 μm)	✓ (150 nm)	✓ (atomic)	✓ (12 μm)
High yield	✓	✓	×	✓
Low cost	✓	✓	×	✓
GO-free	×	×	×	✓
Direct control of surface morphology and properties	×	✓	×	✓

Since LIG’s discovery, tremendous research efforts across the globe have been paid to improve the synthesis of LIG and transiting it into a plethora of application areas. For the synthesis, the precursors have been extended from PI to almost all kinds of substrates such as various commercial polymers [3, 25], metal/plastic composites [26, 27], and naturally occurring materials [12, 28]. In addition, LIG can be easily embedded in other host materials to form functional composites [29, 30], which improves the mechanical flexibility and stretchability. The scale-up manufacturing of LIG, such as laminated printing and roll-to-roll production could be achieved via the optimization of laser settings and the design of an automation streamline [31–33]. The advances in LIG synthesis and engineering have expanded its use in diverse fields. For example, the high-performance micro-supercapacitors based on LIG could be attained by engineering efforts such as series or parallel configuration [34] and chemicals pathway such as heteroatom doping or making a composite [27, 35, 36]. The self-sterilizing property of LIG was studied for water treatment [29, 37, 38]. A variety of chemical reactions, such as oxygen reduction reaction [26], oxygen evolution reaction [28], and hydrogen peroxide generation [38], could be catalyzed by the metal/LIG and metal oxide/LIG composites.

In addition to the above-mentioned applications, the ability to control the shape of LIG and the excellent properties of LIG have made it a power technique in developing highly sensitive and robust sensors for the detection of a diversity of stimuli. The development of highly sensitive sensors is imperative in our daily lives. For instance, the outbreak of coronavirus disease (COVID-19) swept globally and has been designated a global health emergency by the world health organization (WHO) [39]. Almost ten thousand cholera and other water-borne disease cases and the ongoing global warming affect people’s daily life [40]. To tackle these problems, sensors play an essential role. For example, healthcare sensors for monitoring body temperature and respiratory could reflect the conditions of patients. The detection of environmental quality such as air and water are important for maintaining a healthy and safe living environment. Fortunately, with global research efforts, the LIG technique has been developed to detect a broad range of stimuli. This review focuses on the advancement of sensors fabricated from the LIG technology. We first briefly introduce the fabrication and structural modification of LIG. The design, mechanism, and the performance of LIG-based sensors are then summarized in the following section. Finally, we will discuss the impact of LIG and its future development.

## Synthesis of LIG

In this section, we will overview the synthetic efforts for the synthesis of LIG and its modifications pertaining to the fabrication of LIG-based sensors.

### Fabrication and Engineering of LIG

**Fig. 1:**
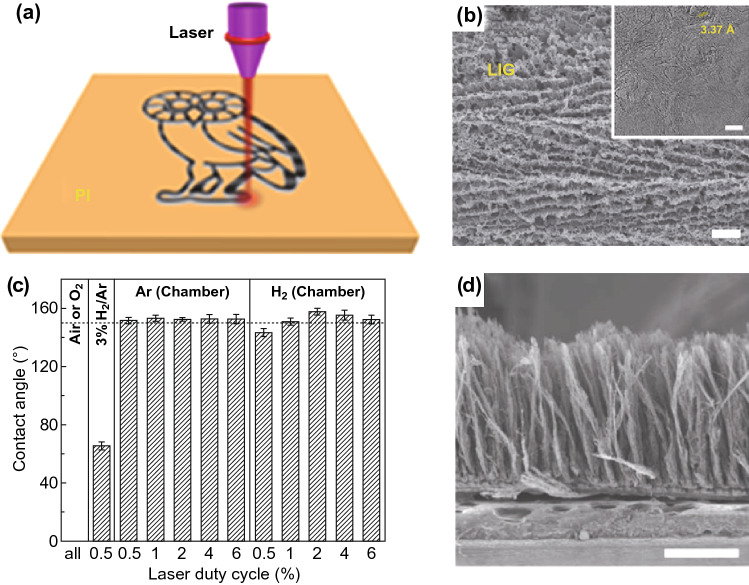
Tunable structure and compositions of LIG. **a** Schematic of the synthesis process of LIG from PI. **b** SEM and HRTEM (inset) image of LIG, scale bar is 10 μm and 5 nm, respectively. **a**, **b** Adapted with the permission from Ref. [3], Copyright 2014 Springer Nature. **c** Contact angles of LIG samples prepared under different gas atmospheres with different laser duty cycles. Adapted with the permission from Ref. [41], Copyright 2017 WILEY-VCH Verlag GmbH & Co. KGaA, Weinheim. **d** SEM image of LIG fiber, scale bar is 500 μm. Adapted with the permission from Ref. [42], Copyright 2018 Elsevier

In 2014, Jian Lin found that the PI could transform into porous graphene when it was lased by a CO_2_ laser in ambient conditions [3]. The shape of graphene, as demonstrated by the “owl” shaped LIG in Fig. 1a, was controlled by the programmable computer design without using a mask. The scanning electronic microscopy (SEM) and high-resolution transmission electron microscope (HRTEM) in Fig. 1b show graphene’s high porosity and characteristic lattice space of ~ 3.4 Å. With the control of atmospheric compositions, the group tuned the surface properties of LIG with a contact angle ranging from 0° to > 150° (Fig. 1c) [41]. By changing the radiation energy, operational modes, pulses density, and laser duty cycle, the graphene morphology could vary from sheet to fiber (Fig. 1d) and to droplets [42], as well as spherical [25] and tubular structure [43]. The morphic transition helps the manipulation of properties. For example, the LIG changed from hydrophilic to superhydrophobic due to the different surface tension [43]. In addition to PI, other biomaterials or synthetic polymers such as polyetherimide (PEI) [3], wood [12], food [44], and polysulfone (PSU) [37] have also been successfully converted to LIG by multiple stepwise lasing, addition of fire retardant or defocused lasing.

The composition engineering of LIG helps to improve its chemical and mechanical properties. This includes the heteroatom doping, formation of hybrid composites, and the embedded structures. The heteroatom doping of LIG could be achieved by using additives-containing precursor or polymer composites and changing carrier gases in the lasing atmosphere [27, 37]. The LIG hybrid material could be obtained by subsequent deposition of functional materials onto LIG. This can be achieved by electrodeposition [45] or by a second lasing of the metal salts-loaded LIG [35]. The embedded structure was attained by first synthesizing LIG on PI substrate and then infiltrating filler such as PVA and PDMS. After curing, the PI substrate was peeled off and the LIG would be left in the fillers. This embedded structure could greatly improve the adherence between substrate and LIG [29].

### Mechanic Properties of LIG

**Fig. 2:**
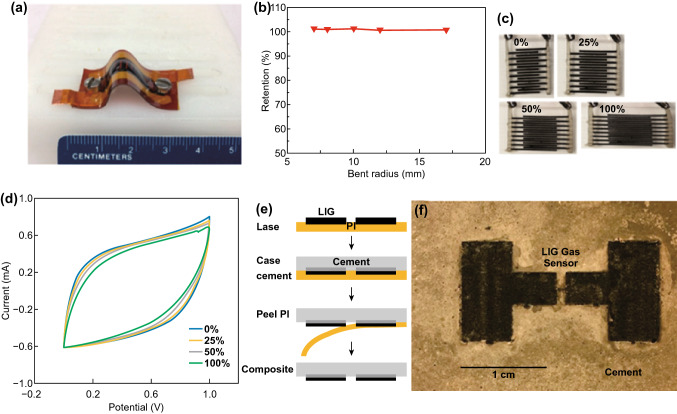
Mechanic LIG and its composites. **a** Digital photograph of a bent boron-doped LIG at a bending radius of 10 mm. **b** Capacitance retention of boron-doped LIG capacitance at different bending radii. **a**, **b** Adapted with the permission from Ref. [27], Copyright 2018 Elsevier Ltd. Images **c** and stretchability test **d** of a S-MSC at 0, 25, 50, and 100% stain. **c**, **d** Adapted with the permission from Ref. [46], Copyright 2019 WILEY-VCH Verlag GmbH & Co. KGaA, Weinheim. **e** Schematic showing the process of embedding a LIG-based sensor into cement. **f** Optical image of the LIG sensor-embedded in cement. **e**, **f** Adapted with the permission from Ref. [47], Copyright 2019 American Chemical Society

The achievement in controlling the structure and composition of LIG has further improved its properties and expanded its use. Sensors working in different environment require different mechanic properties. For example, for wearable electronics, the mechanic flexibility and stretchability should be afforded. Figure 2a, b shows the mechanic flexibility of a LIG supercapacitor fabricated on a PI substrate. Benefiting from the mechanical strength of PI and the integrity of LIG structure, the capacitance retention of boron-doped LIG capacitance still achieved nearly 100% at a bend radius of 17 mm [27]. Figure 2c, d shows the images and stretchability test of a single stretchable micro-supercapacitors (S-MSC) made from LIG composites. S-MSC under different stretching states showed similar capacitive properties and only 15% loss of the initial capacitance after repeating 100% stretching [46]. For sensors used in construction, the mechanic integrity and rigidity are more important for adapting to the surrounding extreme environment. A LIG embedded with cement gas sensor was fabricated by the process shown in Fig. 2e. The cement was well intercalated within the LIG large pores and the LIG pattern kept intact after transferring LIG from PI to cement (Fig. 2f). This system could work under ultrahigh temperature while maintaining the structural integrity [47].

## LIG-Based Chemical Sensors

Chemical sensors are broadly used in the examination of food safety, the contaminants in aquaculture, and portable water, air quality around industries with hazard gas emissions, and the metabolites such as glucose, lactic acid, and dopamine in point of care. The working mechanism of chemicals detection usually relies on the variation in electric signals including resistance, capacitance, and the charge transfer resistance induced by the stimuli. The detection of such variation could be cataloged into two main groups, one is based on the specific binding of chemicals to the surface of LIG, and the other is the non-specific binding detection pathway.

### Specific Binding of Chemical Sensors

**Fig. 3:**
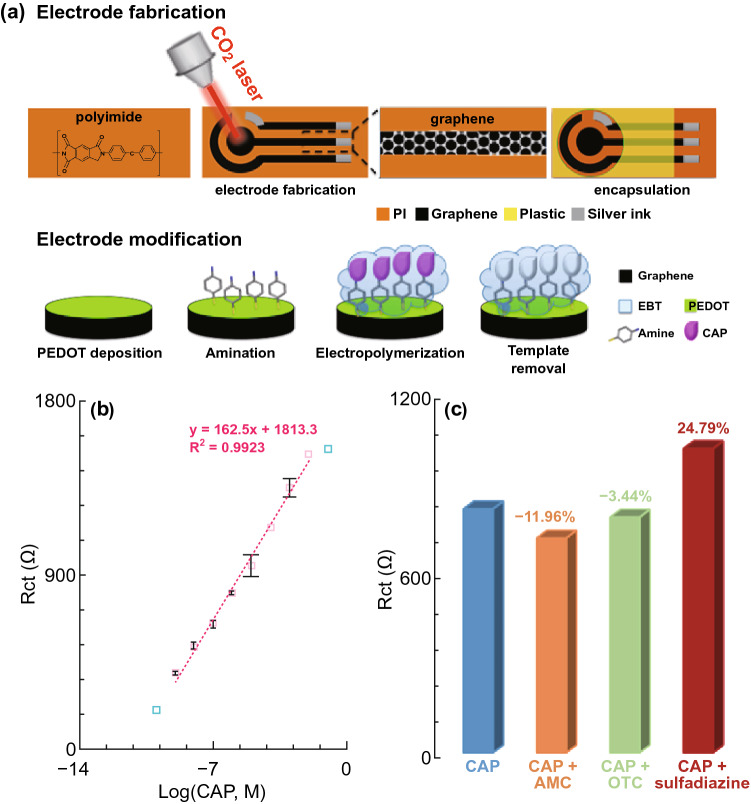
Fabrication process and sensing performance of CAP sensor. **a** Schematic representation of the workflow employed on the production of the LIG electrodes (top) and the MIP fabrication (bottom) for the electrochemical biosensor for detection of CAP. **b** Dependence of *R*_ct_ on CAP concentration. **c** Selectivity behavior of the biosensor for CAP against OTC, AMC and sulfadiazine. **a**–**c** Adapted with the permission from Ref. [48], Copyright 2018 Elsevier B.V

The specific-binding-type chemical sensors are established on the surface functionalization of the LIG with probes such as antibodies (an immunoglobulin which could recognize a unique molecule of the pathogen), enzyme (biological catalysts), and aptamers (a short DNA sequence which can specifically combine with thrombin). Due to the precise combination between recognition elements and targeted chemicals, the sensors often show extraordinary sensing sensitivity. Cardoson et al. prepared LIG electrode combined with a biorecognition element to detect chloramphenicol (CAP) [48]. Figure 3a shows the fabrication of three electrodes by one-step and mask-free LIG technology and the modification of the working electrode. To stabilize the loosen LIG particles and receive more sensing layers, the 3,4-ethylenedioxythiophene (EDOT) was electrochemically polymerized to form PEDOT in the working electrode. The same strategy of the PEDOT deposition was applied in LIG-based dopamine sensor [49], which significantly enhanced the electron transfer responses and the sensing performance. Eriochrome black T (EBT) was electropolymerized in the presence of the CAP template. Molecularly imprinted polymer (MIP) was then formed and served as the recognition element of CAP sensor. The sensor which was assembled without CAP template was referred to NIP. When the concentration of CAP increased, there would be more interaction between CAP and MIP, which interfered with the interaction between the electrode surface and electrolyte. The charge transfer resistance (*R*_ct_), a parameter reflecting the charge transfer between the electrode surface and electrolyte, could therefore be used to measure the concentration of CAP. The analytical performance of CAP sensor was demonstrated in Fig. 3b, c. The MIP maintained the linear behavior at the concentration from 1 nM to 10 mM with an average slop of 162.5 Ω/decade and a limit of detection (LOD) as low as 0.62 nM, while NIP had no specific response with a random relationship between *R*_ct_ and CAP concentration. The different response behavior between MIP and NIP underlined the key role of specific binding. In addition, the selectivity of MIP was studied by interfering with oxytetracycline (OTC), sodium sulfadiazine, and amoxicillin (AMC). The low value of relative standard deviation (RSD) for interference species OTC and AMC indicated the excellent selectivity of MIP. Though RSD for sodium sulfadiazine reached 24.79%, it was because of the chemical reaction between CAP and sulfadiazine rather than the interfering effect of sensing surface. The response sensitivity and selectivity of LIG-based MIP sensor were comparable with sensors made by commercial graphene- and carbon-based screen-printed electrode. The work showed a high potential of printable LIG-based MIP sensor for on-site analysis.

**Fig. 4:**
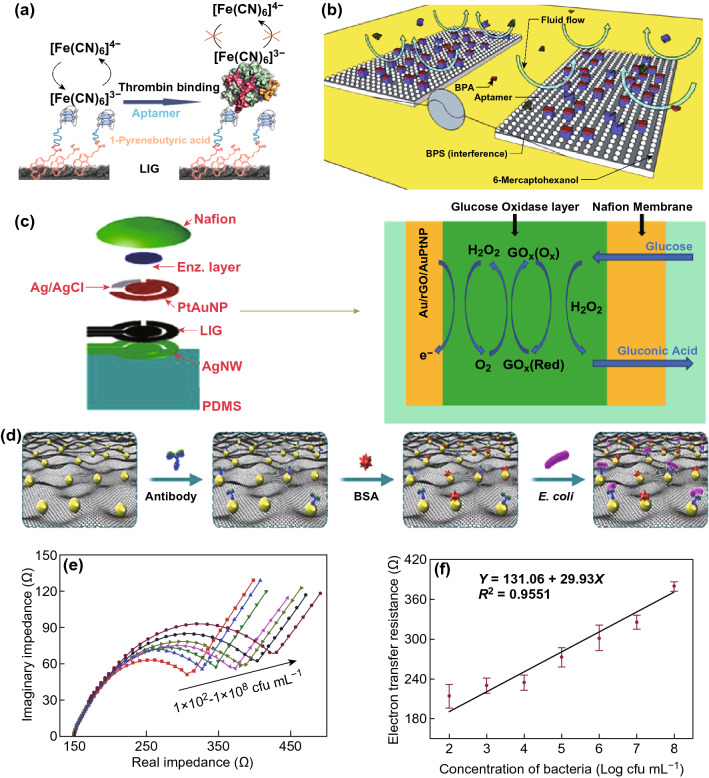
Various specific binding sensors. Schematic of **a** thrombin sensor, **b** BPA sensor, and **c** enzymatic glucose biosensor sensor. Adapted with the permission from **a** Ref. [50], Copyright 2017 American Chemical Society; **b** Ref. [51], Copyright 2016 American Chemical Society; **c** Ref. [52], Copyright 2018 Elsevier B.V. **d** Schematic illustration of the AuNPs-LIG-based immunosensor for the detection of *E. coli* O157:H7. **e** Nyquist plots of *E. coli* sensor. **f** Calibration curve of the impedance response with the concentrations. **d**–**f** Adapted with the permission from Ref. [60], Copyright 2019 Elsevier B.V

Using similar specific binding mechanism, a host of materials have been successfully detected, ranging from small molecules to biomolecules and even pathogen. For example, small molecules such as thrombin (an enzyme emerging in clotting process that promotes platelet activation and aggregation) [50] and bisphenol A (BPA) [51] were detected by immobilizing specific aptamers onto LIG. Glucose [52], biogenic amines [53], and urea [54] sensors were fabricated by anchoring enzymes, and the recognition of ions and the measurement of concentrations were achieved by the functionalization of ionophores [55]. These sensors are based on the change of surface properties after interacting with the chemicals, which can be transduced into electric signals ranging from surface capacitance to redox current densities, and resistance. Figure 4a illustrates the assembly process of aptamer functionalized LIG electrode for thrombin detection using the redox current density. The functionalization of 1-pyrenebutyric acid (PBA) on LIG provides electrode enough carboxyl groups, which could be rapidly and covalently bonded with the amino-functionalized aptamer. According to the differential pulse voltammetry (DPV), the bare LIG electrode without PBA modification showed almost no change before and after aptamer functionalization, underlining the important role of PBA. LIG possesses high specific surface area, it has a large number of edge plane/defect sites and high heterogeneous electron transfer rate. Potassium ferricyanide (Fe (CN)_6_^3−/4−^) was then used as the inner-sphere redox species to indicate the surface property of electrodes [56, 57]. In short, the peak current of redox couple decreases with the reduction in edge plane content of electrodes. The increase in thrombin concentration leaded to the reduction in peak current, which was because that the thrombin captured by aptamer reduced the edge plane area and decreased the heterogeneous electron transfer rate of Fe (CN)_6_^3−/4−^ [50]. As a result, the higher thrombin concentration, the less LIG electrode surface was available for hexacyanoferrate (III), and thus, the lower peak current rendered in DPV. Besides the redox signal as used by the thrombin sensors, the variation in surface capacitance of LIG upon specific binding is another effective mediator for detection. This was shown by Cheng et al., who developed a LIG sensor for the detection of BPA (Fig. 4b) [51]. When BPA bonded to aptamer, as the BPA particles are non-conductive, it inhibited the interfacial charge accommodation and hence reduced the capacitance. The authors also found that introducing alternating current can speed up the transportation of BPA molecules, which significantly curtailed the response time. The superhigh sensitivity of this BPA sensor were ascribed to the porous nanostructure of LIG and the specific binding between aptamer and BPA. A third type of specific detection methods is from the catalytic reaction of enzyme. Figure 4c shows the detection mechanism of an enzymatic glucose sensor from cascade reactions [52]. Ag/AgCl and LIG (rGO) served as reference electrode and working electrode, respectively. The deposition of silver nanowires (AgNW) on LIG was to improve the conductivity of LIG under mechanical deformation. The filtration of PDMS was for further peeling off electrodes from PI. And the additional Au and Pt nanoparticles (AuPtNP) were used as the catalysts to greatly increase the electrochemically active properties and deformability. At the presence of glucose, the glucose oxidase (GO_X_) will produce gluconic acid and hydrogen peroxide (H_2_O_2_). The generated H_2_O_2_ will then be detected by the LIG working electrode from the amperometric current response induced by the oxidation reaction of H_2_O_2_. From the current density, the glucose concentration could be reflected [58]. The glucose could be detected with high sensitivity and not affected by the addition of ascorbic acid (AA), uric acid (UA), and NaCl solution, as the glucose oxidase interacts specifically with glucose. The detection of glucose could also be achieved using other sensing elements such as fluorescent probes [59], which also show high selectivity and sensitivity in detection. Yet the LIG sensors might have advantages in certain scenario as it does not require specific instrumentation. Other analytes, such as urea [54], can also be selectively monitored by using their corresponding enzymes.

In addition to small molecules and biomolecules, the detection of pathogen from the variation of electrode impedance was reported by Wang’s group [60]. The antibody and bovine serum albumin (BSA) were anchored onto LIG for the specific absorption of pathogen *E. coli* O157:H7 (Fig. 4d). When *E. Coli* covered the LIG surface, it interfered with the charge transfer between the electrode and the electrolyte and increased the resistance. Therefore, as the concentration of *E. coli* ranged from 1×10^2^ to 1×10^8^ cfu mL^−1^, the semicircle diameter of Nyquist plots increased and a linear relationship between the *E. coli* concentration and the electron transfer resistance was found (Fig. 4e, f). Yet the non-target bacteria had no significant response. The author also compared different electric signals induced by the adsorbed *E. coli* and found that the charge transfer resistance had a much higher detection sensitivity than sheet resistance and double layer capacitance. Insignificant impedance change of ≤ 10% after hundreds of bending cycles confirmed the excellent flexibility of the LIG-based pathogen sensor.

### Non-specific Binding of Chemical Sensors

Non-specific binding chemical sensors also play an important part in chemical sensors. Without the use of recognition elements such as antibody and aptamer, the cost of the non-specific binding sensors is usually lower. Both the intrinsic chemical redox reactions and the physical properties of the chemicals are informative sources for sensing.

#### Chemical Redox Reaction

**Fig. 5:**
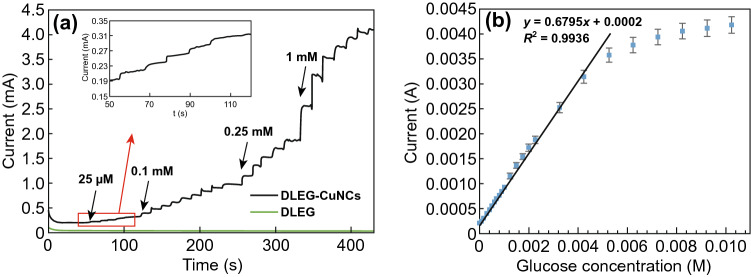
**a** Amperometric current response with successive addition of different glucose concentrations. **b** Calibration curve of the glucose sensor. **a**, **b** Adapted with the permission from Ref. [62], Copyright 2016 Springer Nature

The chemical redox reaction has been commonly used for the detection of solutes and even gas molecules. The detection could be both qualitative and quantitative. For example, the redox potentials help to differentiate different analytes, and the current density related to the redox reaction can provide information on the concentrations of analytes. Gao’s group reported a wearable sensor for uric acid (UA) and tyrosine (Tyr) detection in sweat [61]. DPV is capable to evaluate different analytes by extrapolating information from the oxidation current peak intensities and oxidation potentials. The oxidation peaks of UA and Tyr located at ~ 0.39 and ~ 0.64 V, respectively, which simultaneously detected different metabolites. Tehrani and Bavarian fabricated a disposal glucose sensor using direct laser engraved graphene (DLEG) with decomposition of copper nanocubes (CuNCs) [62]. When added glucose with different concentration, the current increased with different amplitude (Fig. 5a), showing the feasibility of quantitative detection. Figure 5b illustrates the current were in linear relationship with the glucose concentration, and the excellent sensitivity of 4532.2 µA/mM/cm^2^ and linear range from 25 µM to 40 mM were achieved. Non-enzymatic H_2_O_2_ sensor [63] and dopamine sensor [64] based on the reduction current and concentration of H_2_O_2_ was also successfully made.

#### Physical Properties

**Fig. 6:**
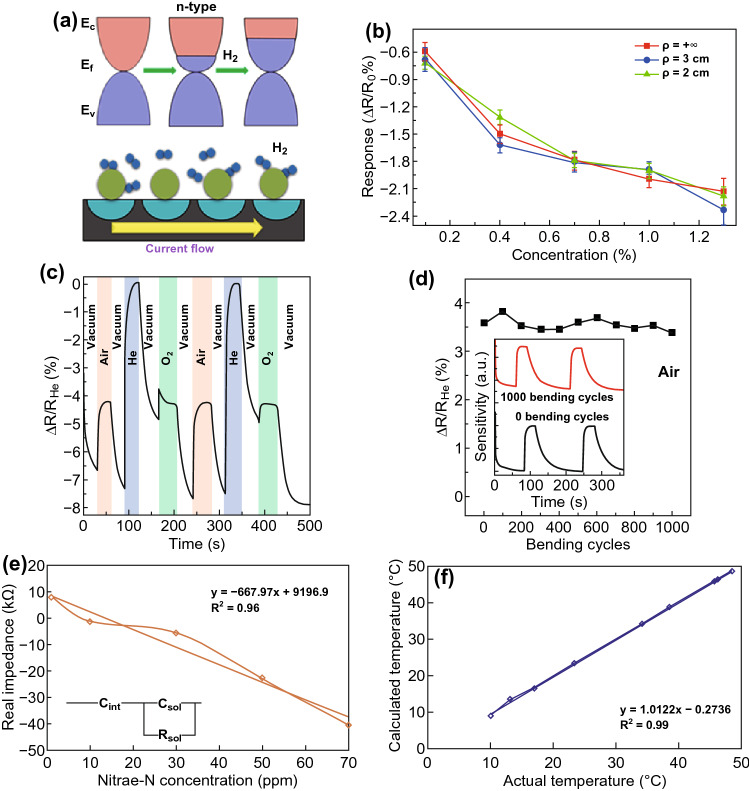
Non-specific binding sensors from the intrinsic and extrinsic properties. **a** Band energy analysis of the H_2_ gas acting onto LIG (top) and catalytic reaction of H_2_ on LIG/Pd (bottom). **b** Response versus H_2_ concentration with different bending states. **a**, **b** Adapted with the permission from Ref. [65], Copyright 2019 American Chemical Society. **c** Responses of gas sensor toward a variety of gases. **d** Magnitude of response of gas sensor to air after bending it with a radius of curvature of 7 mm. Inset figure shows the response of the gas sensor to air after 0 and 1000 bending cycles. **c**, **d** Adapted with the permission from Ref. [47], Copyright 2019 American Chemical Society. **e** Nitrate sensor response to the nitrate concentration. Inset is the equivalent circuit of sensor immersed in solution. **f** Comparison of actual and measured temperature. **e**, **f** Adapted with the permission from Ref. [68], Copyright 2017 Elsevier B.V

The physical properties such as the resistance of LIG upon interacting with analyte and the conductivity or impedance of analyte solution are also used to probe the response from stimuli. For example, an artificial nose based on the chemical bonding between palladium (Pd) and hydrogen (H_2_) for hydrogen detection was made by the Park group [65]. The turbinate plays an important role for odor perception due to the large surface area nature and the ability to propel air toward the olfaction nerve receptors. Inspired by the turbinate structure, biomimetic turbinate-like LIG-based H_2_ sensor was developed. The sensor made use of LIG’s high porosity and electric conductivity, which helped to improve the sensitivity of the device. The Pd nanoparticles (NPs) were used as the medium for hydrogen sensing because of the high affinity of hydrogen to Pd. Figure 6a illustrates the catalytic reaction mechanism of LIG/Pd senor. The as-prepared LIG showed n-type behavior due to considerable oxygen and nitrogen atoms on LIG. The absorption of H_2_ by Pd NPs changed the Fermi energy level of Pd and reduced the work function of Pd. The charges then transferred from Pd to LIG, and thus, the charge carrier density of n-type LIG increases, leading to the decrease in LIG’s resistance. The resistance varied with H_2_ concentration linearly. The authors further transferred the LIG/Pd composites into flexible polyethylene terephthalate (PET) substrate and measured the resistance response under different bending states (Fig. 6b). The negligible variation in resistance response under different bending strength evidenced the excellent mechanic flexibility of the H_2_ sensor. Similar working mechanism was employed in NO_2_ detection by Ho group [66].

The thermal conductivity of gas is another useful parameter for the fabrication of gas sensors, as reported by Tour’s group [47]. The sensor was fabricated by linking a LIG filament with a width of 57 µm to two planar LIG electrodes (Fig. 2f). When the device was Joule-heated, most of heat localized around the filament because of its large resistance. When the sensor was exposed to gas, the heated filament cooled down due to the convective heat loss to the gas. Gas with higher thermal conductivity decreases the temperature of filament more significantly. Since the resistance of the filament is temperature-dependent, the variation in resistance would therefore help to identify the gas. The katharometer-like gas sensor could be used to monitor various gas once the thermal conductivity, and the temperature relationship of tested gas was unequivocal. Various gases such as air, helium, oxygen, and carbon dioxide have been detected (Fig. 6c). Figure 6d shows response of air sensor bending with a radius of curvature of 7 mm within 1000 cycles, and the minor variations implied that the LIG-based gas sensor possessed robust response and good mechanic flexibility. In addition, the author embedded the gas sensor into cement and demonstrated the viability of using this smart building material for the monitoring of the compositions in flue gas.

There are also other non-specific binding chemical sensors built on the extrinsic properties of analytes. For example, Nag’s group exploited salinity (sodium) sensor [67] and nitrate sensor [68] from the resistance of the solution. The impedance of a solution consists of internal capacitance (*C*_int_), resistance of solution (*R*_sol_), and capacitance of solution (*C*_sol_). *R*_sol_ and *C*_sol_ are influenced by the solution medium. The real part of impedance *R*_sol_ was used to investigate the ion concentrations. When the concentration of solution increased, the *R*_sol_ reduced due to the enhanced ionic conductivity. Figure 6e depicts the linear impedance response toward nitrate concentration, and the sensor achieved a wide detection range of 1–70 ppm. Since the ionic conductivity could also be affected by temperature, the author further added a LIG-based temperature sensor to correct the temperature effect. The temperature sensor was designed from the same mechanism as the Tour’s [47], which was based on the correlation between the resistance of LIG and the surrounding temperature. Figure 6f shows that the measured temperature from the LIG-based sensor was consistent with the actual temperature. The compensation of temperature interference greatly improved the precision of sensing. The LIG-based humidity sensors also utilized the extrinsic properties (change of capacitance) [69, 70]. Although extrinsic properties of analyte provide a simple detection pathway, this type of chemical sensor usually has inferior accuracy and precision when compare to the specific-binding sensors and the non-specific-binding ones based on the intrinsic and characteristic chemical and physical properties of analytes. For example, ion concentration sensor will be interfered by other ions in a complex system where there are all sorts of chemicals rather than a single species.

## LIG-Based Mechanic Sensors

**Fig. 7:**
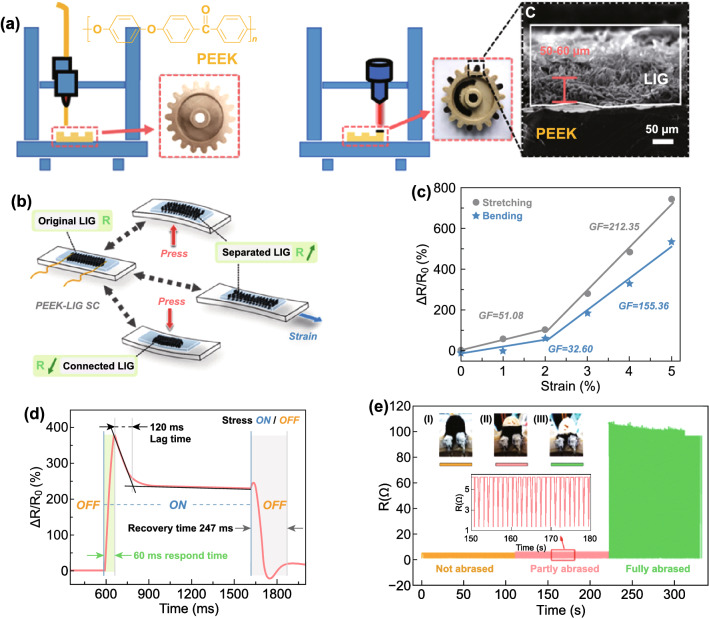
**a** Schematic of the 3D printing of the PEEK component and the synthesis process of LIG from the 3D printed PEEK gear. **b** Working mechanism of PEEK–LIG SC for bidirectional bending and stretching. **c** Relative change in resistance of the sensor versus the applied strains. (The data were obtained after more than 1000 unloading cycles for the bending and stretching.) **d** Respond time and recovery time for bending (0–5% strain). **e** Circuit resistance increases because of abrading of the gear. The inset photographs showed three different abrasion degrees of the smart gear. (I) Not abrased, (II) partly abrased, and (III) fully abrased. **a**–**e** Adapted with the permission from Ref. [71], Copyright 2019 American Chemical Society

Mechanic sensors are widely used in subtle human motion detection, sign language translation, and soft robotic gripper. The LIG-based mechanic sensors are usually built on the piezoresistive effect, which detects the change of resistance due to the shape deformation induced by the stimuli. For example, Zhao’s group combined the 3D printing technique with the LIG process to fabricate smart components (SC), which helped to reflect the conditions such as the working process and abrasion (Fig. 7a) [71]. With computer-control design, they fabricated smart gear from polyetheretherketone (PEEK) with LIG patterns. The PEEK-LIG SC responded to both the bending and stretching of PEEK components, as shown in Fig. 7b, c. The resistance response of strain sensor was correlated with the connection and compactness of LIG on PEEK. When the SC was bended outward or stretched, the resistance increased due to the a loosened connection of LIG. In contrast, bending inward densified the LIG and therefore reduced the resistance. The gauge factor (GF) was 212.35 and 155.36 for stretching and bending, respectively, which suggested a higher sensitivity of planar strain. The response time and recovery time were short (Fig. 7d), which was ascribed to the high elasticity modulus of PEEK. As shown in Fig. 7e, the resistance of gears was correlated with the conditions of the LIG. When the gear was abrased, the resistance increased accordingly. The proposed smart gear could detect its rotation and abrasion while it was working, showing great promise for self-monitoring systems.

**Fig. 8:**
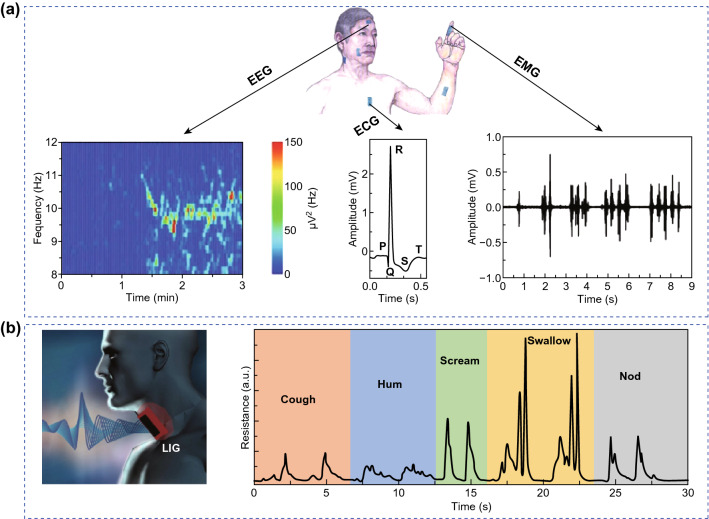
**a** EEG, ECG, and EMG measurements. Adapted with the permission from Ref. [72], Copyright 2018 WILEY-VCH Verlag GmbH & Co. KGaA, Weinheim. **b** LIG-based artificial throat with sound-sensing. Adapted with the permission from Ref. [7], Copyright 2017 Springer Nature

By recording the piezoresistive effect chronologically, LIG-based mechanic sensors can be used for the in situ detection of a variety of stimuli such as heartbeat, motions, and sounds. For example, by attaching the LIG mechanic sensors to different locations of human body, Lin’s group has successfully detected different electrophysiological processes such as electroencephalograms (EEGs), electrocardiograms (ECGs), and electromyograms (EMGs) [72]. The mechanic sensors were made by transferring LIG into an elastomer in a kirigami design, which improved the stretchability of the devices. As shown in Fig. 8a, alpha rhythm with frequency centered at 10 Hz from sensors on forehead implied that the brain waves were successfully recorded. The characteristic P-wave, QRS complex, and T-wave of ECG were identified clearly. And the EMG signals responded to finger bending, which can be used for human–machine interface application. Tao’s group reported the fabrication of a LIG-based artificial throat for sound sensing (Fig. 8b) [7]. When the throat was attached with a LIG sensor, the vibration of throat cords changed the resistance of LIG synchronously. As different sounds generated different wave shapes of resistance, by recording a database and combining with machine learning, the recognition of sound was attainable. The report of LIG-based sound source was also found in the literature [73, 74]. With similar detection principle, some groups reported the improvement in the performance of LIG-based piezoresistive sensors by modifying the structure and composition of the devices. For example, Luo et al. found that the laser conditions dictating the morphology and structure of LIG had great effect on piezoelectric sensor’s performance, and the optimized LIG sensor showed higher gauge sensitivity than commercial strain gauge by nearly 10 times [75]. Chhetry and co-workers designed a MoS_2_/LIG strain sensor for the detection of voice, eye-blinking, and pulse wave [76]. The decoration of MoS_2_ significantly reduced the crack in LIG and improved the mechanical strength of the sensor. By replacing the PI film with a PI paper as the substrate for LIG synthesis, Wang et al. improved the homogeneity and integrity of the LIG and demonstrated the application as a strain sensor to capture the motions of human finger and soft robot [77]. Utilizing the mechanical and acoustical performance of LIG, Tao et al. fabricated a dual-functional device for physiological signals (wrist pulse and respiratory) detection, and self alarming, which offers a brand new idea for health monitoring sensors [78].

## Summary and Outlook

Since the discovery of LIG in 2014, the advances in synthesis of LIG technology have significantly improved the properties of graphene and added to the versatility of applications. For instance, the wavelength of laser extends from infrared to visible and even ultraviolet, which helps to improve the spatial resolution of the LIG structure to ~ 12 µm [6]. The strategies for the formation of LIG composites, such as the in situ and ex situ modification process, can enhance the physical properties of LIG such as mechanical strength and conductivity, as well as the chemical properties by incorporating functional materials [32, 33].

The low cost of LIG technology and simplicity in synthesis promotes the development of a serial of LIG sensors and makes it a potential candidate for industrial production. With the rational design of sensing mechanism, a large diversity of stimuli has been detected ranging from various chemicals to sounds, motions, and temperature. These sensors often show high sensitivity and high stability due to the high surface area and chemical stability of LIG. In addition, the high conductivity of LIG makes it an ideal transducer for converting the stimuli into electrical signal. Pristine LIG made from polymers is often flexible, and its transfer to other substrates such as elastomers or cements could confer it stretchability or rigidity, which makes LIG feasible for use in different scenarios such as wearable electronics and smart building.

The development of LIG sensors has evolved from a single detection component into integrated systems. Real-time and continuous detection of stimuli has been achieved by the integration of wireless transmission and microcontroller modules with sensors for Internet of Things (IoT) applications [47, 65, 67, 79]. For example, Gao’s group incorporated flexible printed circuit board (FPCB) and microcontroller with LIG-based UA and Tyr sensor [61]. This integrated system can wirelessly record sensing signals and convert the digital signal to analog output, which paves a way for the in situ and noninvasive monitoring of health conditions. The Brukitt group assembled the sensor with smart microcontroller system and wireless connection, which helps to form a distributed sensing network for the real-time monitoring of the water quality [68].

Being a patternable and printable manufacturing technique, the LIG-based sensors illuminate a new pathway for developing integrated miniaturized devices. Yet there are still some rooms for improving the LIG technology for practical applications. For example, the bonding of LIG layer and precursor substrate is not strong enough in some scenarios. Though circumvention of LIG such as its functionalization with viscous polymer or transferring the LIG to elastomer can resolve such issue, the consumption of chemicals and additional manufacturing steps is not desirable for production. Some proposed LIG sensors were not demonstrated for in vivo or on-site detection, which might not reflect the feasibility, stability and durability of the sensors in real situations. This, however, is important for practical applications, as interferences from the environment and the variation in conditions from laboratory could potentially affect the sensitivity and reliability of the sensors. Nonetheless, with research efforts from the globe, the diversity of the transitions of LIG into various sensors has been rewarding and delightful to behold. With future development, LIG sensors will find a commonplace in widespread applications.
